# Peptide-Based Approaches for Pain Relief and Healing in Wounds

**DOI:** 10.3390/ijms27020685

**Published:** 2026-01-09

**Authors:** Klaudia Kołodyńska, Wojciech Kamysz, Patrycja Kleczkowska

**Affiliations:** 1 Department of Inorganic Chemistry, Faculty of Pharmacy, Medical University of Gdańsk, 80-416 Gdansk, Poland; klaudia.kolodynska@gumed.edu.pl (K.K.); kamysz@gumed.edu.pl (W.K.); 2 Institute of Outcomes Research, Maria Sklodowska-Curie Medical Academy in Warsaw, 03-411 Warsaw, Poland; 3 Department of Biomedical Research, National Medicines Institute, 00-725 Warsaw, Poland

**Keywords:** wound healing, peptides, analgesics, efficacy, challenges

## Abstract

A wound has been defined as a disruption of tissue integrity. Pain, bleeding, and the risk of infection are inherent features of wounds, while chronic wounds are often accompanied by serous exudate. Pain associated with chronic wounds is usually underestimated and inadequately addressed in routine clinical care, despite being considered by patients as one of the most burdensome factors affecting their quality of life. Traditionally, management of wound-related pain has relied primarily on systemic analgesics, commonly administered orally. However, recently, there has been accumulated interest in the potential of topical analgesics. Unfortunately, both systemic and local administrations of conventional analgesics (e.g., NSAIDs, opioids) might carry risks of adverse effects, including delayed wound healing and systemic absorption. In this review, we summarize current research on the use of local analgesia for painful wounds and explore the potential of topically applied peptides with analgesic activity as a promising alternative to conventional pain management strategies. We also discuss recent innovations in the development of therapeutic peptides, including those with anti-inflammatory and regenerative activities, which might further enhance outcomes in the wound healing process. Finally, we address challenges associated with topical peptide delivery across compromised skin barriers and examine strategies to overcome these limitations, while outlining future directions for formulation and clinical application of peptide-based wound therapies.

## 1. Wounds: Classification, Healing and Clinical Assessment

A wound has been defined as a disruption of tissue integrity or the compromise of its physical cohesion, which can occur in the skin, mucous membranes, or internal organs [1,2,3,4,5]. Untreated or improperly managed wounds can lead to serious complications such as infections, chronic ulcers, tissue necrosis, and, in severe cases, life-threatening conditions [6,7]. However, with reference to wound infection, several additional factors can play a role. These include patient-specific characteristics, the quality of the wound itself, and the surrounding environment. Key patient-related factors are systemic conditions such as poorly controlled diabetes, peripheral neuropathy, disorders causing tissue hypoxia, connective tissue diseases, malnutrition or obesity, alcohol or illicit drug use, smoking, corticosteroid therapy, and inadequate adherence to treatment protocols. Wound-related aspects, such as size, depth, location, and level of contamination, as well as environmental factors like hygiene and care practices, can also significantly influence infection risk [8,9,10]. Accurate diagnosis of the wound type, assessment of infection risk, and selection of appropriate therapeutic intervention are therefore essential for effective treatment and prevention of complications.

Wounds can be classified based on several criteria. One commonly used approach is based on the mechanism of injury, distinguishing between mechanical, chemical, thermal, and ulcerative wounds [11]. Other important factors include wound depth (abrasions, superficial (partial-thickness and full-thickness), deep, and penetrating wounds), surgical cleanliness (clean, clean–contaminated, contaminated, and infected), and healing duration (acute vs. chronic) [12,13,14]. Understanding the wound type is crucial for selecting a suitable treatment strategy, as different wounds require specific therapeutic approaches to achieve optimum healing.

**Figure 1:**
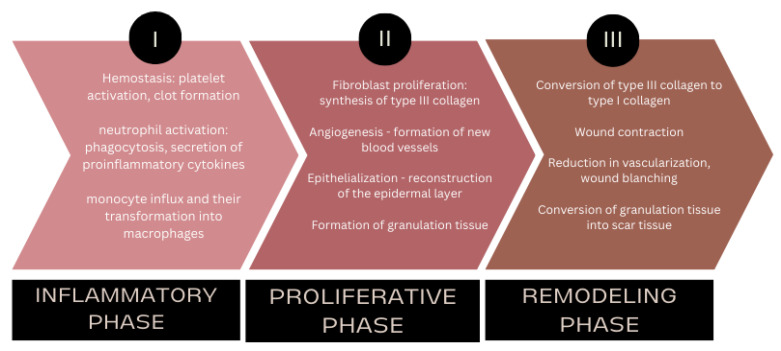
Phases of wound healing. Created in Microsoft PowerPoint, 2021.

The wound healing process itself is complex and heterogeneous, relying on a coordinated interplay of inflammatory mediators, blood components, extracellular matrix elements, and connective tissue cells [15]. The process begins with the inflammatory phase (Figure 1), which starts immediately after injury and is characterized by increased blood flow to the wound and surrounding tissue, localized swelling, and a rise in temperature [16]. Hemostasis is achieved through local vasoconstriction and platelet aggregation, which activate the coagulation cascade (e.g., factors XII, IIa, and Xa) and lead to clot formation. Activated platelets release a variety of factors that initiate subsequent repair stages, while neutrophils and macrophages remove debris and pathogens, preparing the wound bed for regeneration [17,18].

Following the inflammatory phase, the proliferative phase (that lasts from 3 days up to 2 weeks following skin injury) occurs, encompassing angiogenesis, granulation tissue formation, collagen deposition, and re-epithelialization [19,20]. These processes occur in parallel and contribute to reconstruction of tissue structure and function. After several days in acute wounds, or even several months in chronic wounds, the remodeling phase begins. During this final phase (phase III), granulation tissue is reorganized into mature scar tissue, collagen type III is gradually replaced by collagen type I, and the wound regains the mechanical strength and visual characteristics of healthy skin [21,22].

While most acute wounds heal within days to a few weeks, chronic wounds often remain in a prolonged inflammatory state, exhibiting elevated protease activity and impaired progression through these healing phases [23,24,25].

Chronic wounds—including venous and diabetic ulcers, pressure injuries, and thermal burns—represent a significant challenge in clinical practice due to their persistent nature, complex care requirements, and high treatment costs. Management of chronic wounds often requires long-term care with specialized dressings, advanced equipment, and a multidisciplinary approach involving physicians, nurses, pharmacists, dietitians, and physiotherapists. Successful treatment depends on a thorough clinical assessment, which includes evaluation of tissue condition, infection and inflammation status, exudate characteristics, wound edges, surrounding skin, and pain. Tissue assessment helps determine the current stage of healing, while the presence of infection—often indicated by changes in odor, color, or consistency of exudate—can disrupt healing and pose a threat to the patient’s health. Exudate itself is a natural component of the healing process; clear, odorless, and watery exudate at low volume supports tissue repair by maintaining a moist environment conducive to cell migration and epithelial formation [26]. In contrast, copious, thick, discolored, or malodorous exudate, potentially containing pus or blood, indicates abnormalities in healing such as infection or hypergranulation, which require prompt intervention.

Assessment of wound edges and surrounding tissue provides additional insight into healing status. Raised, rolled, reddened, or darkened wound edges might reflect delayed healing or pathological changes, while dryness, flaking, redness, tension, shininess, or pigmentation changes in adjacent skin can signal abnormal tissue responses [27]. Pain, arising from the underlying disease process, surgical trauma, or wound care procedures, can also indicate healing disturbances, infection, or inadequate wound management [28,29]. Systematic evaluation of pain before, during, and after dressing changes allows clinicians to tailor interventions and optimize therapeutic outcomes.

By integrating careful classification, understanding of the healing process, and comprehensive clinical evaluation, clinicians can implement interventions that support tissue regeneration, minimize complications, and enhance patient outcomes. The interconnection among wound type, healing dynamics, and clinical assessment emphasizes the importance of a holistic, patient-centered approach to wound management, particularly in the treatment of chronic and complex wounds, where successful care depends on both the precision of interventions and coordination of multidisciplinary teams.

## 2. Pain as an Integral Component of Wound Pathophysiology

Regardless of the underlying cause, every wound is associated with varying degrees of pain, defined as “an unpleasant sensory and emotional experience associated with, or pretending to be associated with, actual or potential tissue damage” [30].

**Figure 2:**
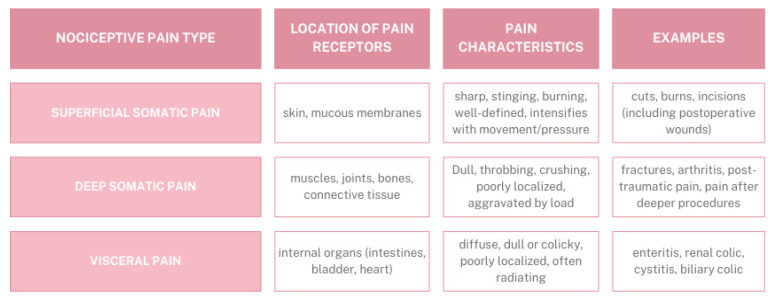
Types of nociceptive pain. Created in Microsoft PowerPoint, 2021.

Different types of pain can be distinguished depending on the classification criterion adopted. The primary classification, based on etiology, differentiates nociceptive pain, neuropathic pain, and nociplastic pain [31]. Nociceptive pain, also referred to as receptor pain, arises from tissue damage due to activation of pain receptors (nociceptors) Nociceptive pain can be further subdivided into deep somatic pain, associated with activation of nociceptors in muscles, tendons, ligaments, bones, or lining tissues such as the peritoneum; visceral pain, occurring within internal organs; and superficial somatic pain, originating from activation of skin nociceptors (Figure 2) [32,33]. Neuropathic pain, on the other hand, results directly from damage or disease affecting the central or peripheral nervous system [33]. While nociplastic pain can be categorized into five main types: chronic widespread pain, chronic primary musculoskeletal pain, chronic primary visceral pain, chronic primary headache, and complex regional pain syndrome [34].

In patients with wounds, nociceptive and neuropathic pain frequently coexist [35]. Pain can arise from direct stimulation of peripheral nociceptors, tissue damage, or edema, which forms part of the inflammatory response [36]. Infection also represents an important source of pain, and any sudden onset, change in character, or increase in intensity should raise suspicion of infectious complications [30].

Wound-related pain can be categorized into several clinically relevant types:

1. Background or baseline pain—caused by the underlying pathology and the wound itself, representing discomfort experienced during routine daily activities.
2. Incidental pain occurs in response to specific movements or activities, such as walking, deep breathing, or coughing.
3. Procedural or operative pain—arises during wound care interventions, including cleaning, dressing changes, or other procedures such as biopsies [37].

Despite its prevalence, pain in wound care is often underestimated and inadequately addressed. Uncontrolled pain significantly diminishes patient quality of life and can contribute to the development of depressive symptoms, sleep disturbances, and activity-related anxiety. Patients frequently report that pain is among the prevailing aspects of living with chronic wounds. Epidemiological data indicate that approximately 80% of individuals with chronic venous leg ulcers experience persistent pain related to their wounds [38,39]. Moreover, a study from 2006 has demonstrated that high levels of perceived pain, whether acute or chronic, could negatively affect the healing of postoperative wounds, highlighting the critical importance of effective pain management in wound care [40].

A comprehensive assessment of wound-related pain should include evaluation of its intensity, duration, nature, and source. Pain assessment is particularly important for selecting effective pharmacological and non-pharmacological interventions during dressing changes and other procedures, as well as during routine daily activities. By recognizing pain as an integral component of wound pathophysiology, clinicians can develop individualized treatment strategies that not only alleviate discomfort but also support optimum healing and improve overall patient outcomes.

## 3. Pharmacological Mechanisms of Analgesics: From Systemic Action to Localized Wound Application

Pain is a multifaceted physiological and biochemical phenomenon resulting from activation of peripheral and central nociceptive pathways. Its modulation is achieved through diverse pharmacological strategies, with agents categorized according to their molecular targets and mechanisms of action. The major classes of analgesics include nonsteroidal anti-inflammatory drugs (NSAIDs), opioid analgesics, local anesthetics, and gabapentinoids, each operating via distinct molecular mechanisms yet converging on the suppression of nociceptive signaling at the site of tissue injury, either along peripheral nerves or within central pain-processing pathways.

In wounded tissue, nociceptive signaling is not merely a consequence of direct activation of peripheral nerves by trauma but arises from a dynamic interplay between immune, inflammatory, and neural components of the tissue microenvironment. Following injury, macrophages and other innate immune cells rapidly infiltrate the wound site, secreting pro-inflammatory cytokines such as TNF-α, IL-1β and chemokines that orchestrate early inflammatory responses and influence both pain and healing processes [41,42]. ATP released from damaged cells and infiltrating immune cells can act as a danger signal and engages purinergic receptors on nociceptors and immune cells, thereby enhancing peripheral sensitization and inflammatory signaling [43,44]. Pannexin-1 channels are increasingly recognized as conduits for ATP release, and experimental blockade of Panx1 has been shown to attenuate peripheral inflammatory pain, underscoring their contribution to nociceptive amplification when inflammation is present [43,45]. In addition to ATP-mediated mechanisms, direct interactions between sensory nerve endings and immune cells further modulate both inflammation and healing: activation of nociceptors results not only in pain transduction but also in release of neuropeptides that regulate the activity of macrophages and other myeloid cells, influencing wound closure and tissue repair [46,47,48]. Collectively, these immune–neural interactions create a pro-nociceptive and pro-regenerative milieu in wounds, setting the stage upon which pharmacological agents act either by dampening inflammatory sensitization or by directly reducing nociceptor excitability.

Among the molecular mediators generated within this inflammatory wound microenvironment, prostaglandins play a central role in peripheral nociceptor sensitization [49]. In this context, NSAIDs, for example, are widely employed owing to their ability to inhibit cyclooxygenase (COX-1 and COX-2) enzymes. This reduces prostaglandin synthesis and suppresses nociceptor activation at the periphery [50,51]. This mechanism underpins both systemic analgesia and emerging strategies for local application, where NSAIDs incorporated into wound dressings or hydrogels can exert anti-inflammatory and analgesic effects directly at the site of tissue injury.

Opioid analgesics act through G-protein-coupled receptors (μ (MOR), δ (DOR), and κ (KOR)) expressed in central and peripheral neurons [52]. In addition, these receptors are expressed in the skin on peripheral nerve endings, keratinocytes, and immune cells such as Langerhans cells and macrophages, providing anatomical basis for local analgesia [53,54]. Receptor activation leads to inhibition of adenylate cyclase, reduction in intracellular cAMP, opening of potassium channels, and closure of voltage-gated calcium channels. Those activities result in neuronal hyperpolarization and suppressed release of neurotransmitters, including substance P and glutamate, thus attenuating nociceptive signal transmission [55,56,57,58]. Peripheral opioid receptors, particularly upregulated during inflammation [59], offer a rationale for topical application in wounds, providing localized analgesia with a minimum systemic exposure.

Local anesthetics, such as lidocaine and bupivacaine, reversibly block voltage-gated sodium channels in excitable membranes, preventing action potential initiation and propagation along sensory neurons [60,61,62,63]. This site-specific blockade produces immediate analgesia without systemic effects, making local anesthetics particularly suitable for incorporation into dressings or hydrogels to alleviate pain during dressing changes or wound debridement. While local anesthetics provide analgesia by selectively suppressing pain perception at the periphery, general anesthetics act centrally to induce loss of consciousness, amnesia, and immobility through modulation of CNS receptors such as GABA-A and NMDA [64,65,66]. Unlike analgesia, which selectively targets nociceptive signaling, anesthesia encompasses a broader suppression of sensory, cognitive, and motor responses.

Gabapentinoids, including gabapentin and pregabalin, bind to the α2δ subunit of presynaptic voltage-gated calcium channels, reducing calcium influx and subsequent release of excitatory neurotransmitters, such as glutamate, noradrenaline, and substance P [67,68,69,70]. By attenuating both peripheral and central sensitization, gabapentinoids are particularly effective in neuropathic pain, which often complicates chronic or inflamed wounds [71,72].

Notably, some pain-relieving drugs also act via additional signaling pathways. The endocannabinoid system, for example, modulates pain through CB1 and CB2 receptors [73]. CB1 receptors, located mainly in the central nervous system, suppress excitatory neurotransmitter release at presynaptic terminals, while CB2 receptors in immune and peripheral tissues regulate inflammatory signaling and cytokine production [74,75,76]. Their stimulation produces both antinociceptive and anti-inflammatory effects, and evidence suggests functional cross-talk with the opioid system, resulting in enhanced analgesic efficacy [77,78].

Nitric oxide (NO) is another important neuromodulator in pain signaling. Beyond vascular [79,80] and immune functions [81,82,83], NO contributes to central and peripheral sensitization by modulating excitatory neurotransmission and interacting with cyclic GMP pathways [84,85,86]. Its dual role, capable of both promoting and attenuating nociception depending on concentration and context [87], emphasizes the complexity of targeting NO-related pathways for analgesic purposes.

The transient receptor potential vanilloid 1 (TRPV1) channel, widely expressed in nociceptive sensory neurons, is another key molecular target. TRPV1 is activated by noxious heat and chemical stimuli [88], and its sensitization is a hallmark of inflammatory pain [89]. Modulation of TRPV can lead to hypersensitivity and hyperexcitability of nociceptor neurons (peripheral sensitization) [90]. Importantly, there is functional crosstalk between NO signaling and TRPV1 activation: NO can modulate TRPV1 activity indirectly through protein kinase pathways, while TRPV1-driven calcium influx influences downstream NO production [91,92]. This bidirectional relationship highlights how multiple signaling systems converge to regulate pain perception and offers novel opportunities for multimodal analgesic strategies.

Interestingly, accumulating evidence indicates that some NSAIDs, such as ibuprofen and diclofenac, exert additional effects beyond cyclooxygenase inhibition. For instance, ibuprofen has been shown to downregulate inducible nitric oxide synthase (iNOS) and reduce NO release during inflammation, thereby indirectly influencing nociceptive signaling [93,94,95]. It can also interact with cannabinoid receptors and, through inhibition of fatty acid amide hydrolase (FAAH), which metabolizes the endocannabinoid anandamide, activate antinociceptive pathways [96].

Nevertheless, localized analgesic delivery in wound care leverages these molecular mechanisms to directly modulate nociceptor activity within the wound microenvironment. Incorporation of NSAIDs, opioids, or local anesthetics into dressings, hydrogels, or nanocarriers enables sustained release, maintaining analgesia while minimizing systemic exposure. This approach allows multiple molecular pathways to be targeted simultaneously, enhancing efficacy and supporting the tissue healing process.

Understanding the molecular and cellular mechanisms underlying analgesic action provides a foundation for rational selection and combination of agents in both systemic and local contexts. Such knowledge is critical for the development of advanced multimodal strategies that integrate pharmacology with wound care technologies, ultimately aiming at improving patient outcomes by optimizing analgesia while preserving tissue integrity.

## 4. Integrated Approach to Wound Pain Management: Pharmacotherapy and Wound Dressings

The management of wound-related pain primarily relies on systemic administration of analgesics such as paracetamol, NSAIDs, opioid analgesics, gabapentinoids (gabapentin, pregabalin), and tricyclic antidepressants (TCAs). However, the efficacy of such a treatment can be limited, as conventional routes of analgesic administration (oral, intravenous, etc.) are often associated with numerous adverse effects. For example, opioids, particularly those capable of crossing the blood–brain barrier (BBB), can induce central nervous system side effects, including sedation, nausea, and risk of tolerance and dependence, which can make patients reluctant to pursue this form of therapy [97,98,99]. Additionally, long-term oral use of NSAIDs can result in renal impairment and gastrointestinal mucosal damage [100,101,102]. Moreover, NSAIDs are contraindicated in patients on anticoagulant therapy due to increased bleeding risk, as well as in those with hypertension [103,104]. Noteworthy, it has been demonstrated that acetaminophen, another type of commonly used nonopioid medication, toxicity can contribute to some opioid-related mortality [105].

An increasingly recognized factor in clinical practice is that wound pain can be significantly exacerbated by inappropriate selection or improper use of dressings, especially during dressing changes [106]. Adhesive dressings that adhere to the wound bed and regenerated tissue are likely to cause trauma and contribute to procedural pain. Therefore, wound management must integrate both pharmacological intervention and optimized local wound care. Appropriately selected dressings serve a dual purpose: they support healing by maintaining a moist environment and protecting the wound from external factors, while simultaneously minimizing pain by reducing secondary injuries and decreasing the frequency of interventions.

Modern advanced wound dressings can exert an indirect analgesic effect by reducing exudate, infection, and friction. Recently, the concept of incorporating analgesic agents directly into wound dressings has gained attention. However, the literature on topical pain management in wounds still remains restricted, with prevailing studies focusing on procedural pain relief during dressing changes. One remarkable study evaluated the use of oxycodone-impregnated dressings in adult patients with third-degree burn wounds [107]. A dressing containing 20 mg of oxycodone prevented pain symptoms, whereas a 10 mg dose significantly reduced pain, with no systemic opioid-related side effects observed.

Numerous studies support the analgesic efficacy of lidocaine in wound care [108,109,110]. Topical application of lidocaine in the form of cream, spray, or gauze soaked in lidocaine solution alleviated pain with relatively mild adverse effects [108,111]. However, lidocaine-soaked gauze often provided insufficient relief, requiring additional anesthesia, and lidocaine aerosol offered only short-term analgesia [111,112,113]. A prospective, double-blind, placebo-controlled trial by Kwon et al. [114] demonstrated significant pain reduction with lidocaine in female patients following laparoscopic gynecological surgery, with no notable adverse effects. In addition, several independent studies have confirmed the analgesic efficacy of EMLA cream (lidocaine + prilocaine) in relieving pain during debridement of venous leg ulcers [115,116,117,118,119]. Furthermore, gelatin sponges soaked in bupivacaine solution placed in postoperative cesarean section wounds also reduced pain and the need for systemic opioids [120].

Oh et al. [121] developed a thermosensitive hydrogel formulation with sustained release of 0.75% ropivacaine, designed to alleviate postoperative pain. Pain relief following application of the hydrogel lasted at least 24 h, with low plasma and tissue concentrations of ropivacaine, indicating minimum systemic distribution. Similarly, a study by Khan et al. [122] evaluated an ointment containing 0.2% glyceryl trinitrate and 2% lidocaine in post-Milligan–Morgan hemorrhoidectomy wounds. This dual-component formulation demonstrated superior analgesic effects and accelerated wound healing as compared to those of single-agent ointments.

Morphine still remains the gold standard in pain management. As one of the most potent opioid analgesics, morphine has long been used to treat pain of varying severity, particularly in cancer, trauma, and postoperative settings [123,124]. Recently, topical morphine-soaked dressings have been investigated to provide targeted analgesia at the wound site while minimizing systemic opioid-related adverse effects [125,126,127]. Although some studies report effective pain reduction [125,128,129,130]. Other studies suggest analgesic effects comparable to those of placebo [126,131,132,133]. Importantly, those studies often involve small cohorts, limiting statistical power and generalizability [134]. For instance, a study evaluating diamorphine gel in advanced pressure ulcers demonstrated significant pain relief but included only seven participants [135].

NSAIDs, including topical ibuprofen, have also been studied, showing reduced pain and improved quality of life in patients with lower leg ulcers [136,137,138,139,140]. An ex vivo study conducted by Russo et al. [141] assessed the rheological properties of a poloxamer-based gel containing sodium diclofenac and its potential application in chronic wound analgesia. The study utilized snake skin dialysis membranes, intact porcine ear skin, and tape-stripped porcine ear skin. Results indicated that the presence of poloxamer allowed for gradual and prolonged release of the active substance through damaged stratum corneum, enabling local analgesic and anti-inflammatory effects of diclofenac with a minimum systemic exposure.

Recently, also, nanoconjugates combining diclofenac with a lipid carrier have been developed [142]. When applied to cryogenic wounds in rats, these formulations accelerated skin and hair follicle regeneration and reduced expression of inflammatory markers (IL-1β, IL-6, TNF-α) and pain-associated markers, including COX-2 (which drives prostaglandin-mediated nociceptor sensitization) [143,144], YKL-40 (associated with inflammation-induced pain signaling) [145], and substance P (a neuropeptide that transmits nociceptive signals) [146], while enhancing expression of angiogenic and remodeling markers (VEGF, TGF-β, FGF, PDGF, MMP-9, EGF) [142].

Older literature reported 3% benzydamine cream as a potential product for painful pressure ulcers, although no statistically significant difference between topical benzydamine and placebo cream was reported [147]. A relatively recent discovery involves sevoflurane, which provides rapid and prolonged analgesia (median duration: 9 h [148]), facilitating advanced wound debridement [149]. Another study compared the analgesic effects of topical sevoflurane with intravenous opioids [150]. Patients treated with sevoflurane reported stronger and longer-lasting pain relief compared to those receiving systemic opioids. Sevoflurane was also found effective in treating pain associated with chronic venous ulcers, additionally reducing wound size, suggesting a potential positive influence on wound healing. Reported adverse effects were mild, including localized itching and burning [150].

Despite the benefits of topical analgesics, concerns exist regarding their impact on wound healing. Diclofenac, for example, might reduce fibroblast proliferation and decrease hydroxyproline levels, impairing collagen synthesis [151,152]. Topical opioids on large wounds carry a potential risk of systemic absorption and central nervous system side effects, particularly in vulnerable populations such as elderly patients, young subjects, or those with renal impairment [153]. Consequently, accessible topical formulations have not been universally recommended for routine wound pain management.

These observations highlight the ongoing need for safe and effective strategies for wound pain control. One promising avenue is the development of analgesic peptides, which are attracting increasing scientific interest owing to their potential to provide localized pain relief while minimizing systemic side effects.

## 5. A New Approach to Wound-Related Analgesia—Antinociceptive Peptides

Peptides are short-chain amino acids exhibiting a wide range of biological activities, including both therapeutic and toxic effects. A specific group of them are analgesic peptides, whose origins date back to the 1970s, when the existence of various subtypes of opioid receptors was confirmed and the first endogenous opioid peptides, enkephalins, were discovered. An increasing understanding of the mechanisms underlying nociceptive signaling has positioned analgesic peptides as promising alternatives to conventional opioid drugs for the treatment of chronic and neuropathic pain, without the risk of tolerance or addiction. Notably, some of these peptides (e.g., biphalin [154]) exhibit not only potent analgesic effects but also support wound healing processes—an additional benefit in the treatment of painful chronic wounds.

A particularly innovative approach in drug delivery technology is the incorporation of analgesic peptides directly into the structure of advanced wound dressings, enabling localized, controlled, and sustained release of the active compounds directly at the wound site. This strategy not only minimizes the necessity of systemic analgesics, which often carries the risk of adverse effects, but also improves therapeutic efficacy by maintaining continuous analgesic activity at the site of injury.

The integration of pain-relieving peptides with wound dressings offers a promising research direction by combining the benefits of advanced pharmacotherapy with modern wound care. Such an approach has a potential to significantly enhance patient comfort and healing outcomes through reducing both procedural and chronic pain and thereby positively improves the quality of life in patients suffering from wounds.

### 5.1. Application and Efficacy of Peptide-Based Structures in Wound-Associated Pain Management: Preclinical Studies

Among the many challenges in wound management, developing therapies that simultaneously promote tissue regeneration and alleviate pain remains a key objective. Currently, a large part of studies on bioactive peptides is focused primarily on their regenerative activities, while the analgesic aspect, especially topical analgesia, is comparatively worked out. Moreover, most preclinical models evaluating the analgesic effects of peptides do not involve wounds as the primary source of nociception. To date, no standardized experimental model has been established that would allow simultaneous assessment of both wound-healing and analgesic efficacy of peptides at the level of injured skin.

Nevertheless, a few peptides have been examined in separate experimental models, demonstrating both antinociceptive and pro-regenerative effects. These are molecules of particularly high translational potential, which can be regarded as candidates for the next generation of bioactive wound dressings.

#### 5.1.1. Peptides with Documented Analgesic and Regenerative Activities

**Figure 3:**
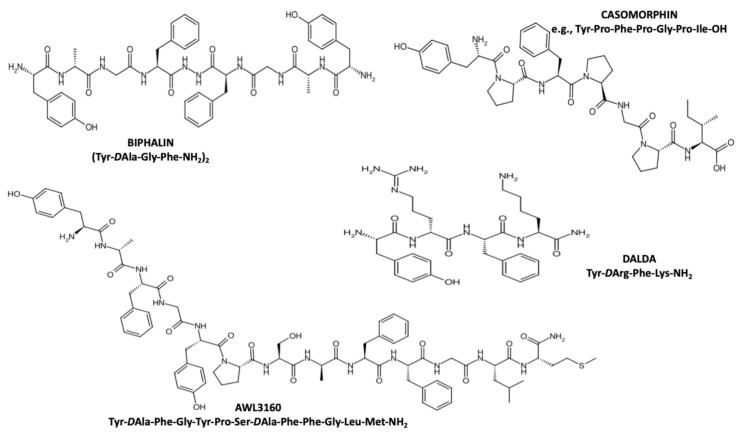
Representative chemical structures of selected opioid-based analgesic peptides studied in animal wound models. Created in ChemDraw Online; https://freechemdraw.com/en/ (accessed on 12 December 2025).

An exemplary compound with high potential in both fields is the synthetic opioid peptide biphalin, developed by Lipkowski in 1982 [155]. Biphalin (Figure 3) is a dimeric octapeptide composed of two tetrapeptides (enkephalin analogs with a D-Ala substituted at the N-terminal position 2), linked by a hydrazide bridge, to form a palindromic sequence. It acts as a potent μ- and δ-opioid receptor agonist, with an EC50 of nearly 1–5 nM for both receptor types [156], providing effective pain modulation with reduced risk of side effects associated with classic opioids [157,158,159]. Additionally, this peptide has demonstrated anti-inflammatory activity in vivo [160,161]. For example, in a corneal injury model, topical administration of biphalin accelerated epithelial regeneration, reduced inflammatory infiltration, and supported structural tissue recovery [162]. More recently, Konop et al. [154] employed a full-thickness dorsal wound model in streptozotocin (STZ)-induced diabetic mice to assess a keratin-based wound dressing incorporating 0.1% biphalin. The dressing significantly accelerated wound healing, both by increasing the rate of re-epithelialization and enhancing granulation tissue formation. Histological analyses revealed increased macrophage infiltration and thicker epidermis in the biphalin-treated group, whereas the control group showed neutrophil predominance. Importantly, biphalin activated the AKT–mTOR signaling pathway, suggesting its role in tissue regeneration and cell proliferation.

In another study [163], hybrid peptides were evaluated for their effects on wound healing in a diabetic rat model. Lanolin-based ointments containing 10% keratin fibers and either biphalin or AWL 3106 (a chimeric peptide combining pharmacophores of dermorphin and fragment 7–11 of substance P; Figure 3) were applied to the wounds. Both peptides shortened the inflammatory phase and reduced wound area, indicating positive regenerative effects.

The peptide DALDA (Figure 3), an amidated derivative of dermorphin D-Arg^2^, Lys^4^, was studied for its analgesic properties in trauma models, but not in combination with wound dressings. In a rat model of post-burn injury pain (Sprague–Dawley) by Modi et al. [164], DALDA effectively alleviated both stimulus-evoked (mechanical, thermal, and cold) and resting pain in a dose-dependent manner (1, 3, and 10 mg/kg s.c.). A dose of 10 mg/kg significantly reduced spontaneous pain without signs of dependency or sedation.

A distinctly different pharmacological profile has been found in LPF-35, a 35-amino acid synthetic analog of lactoferrin [165]. Designed to enhance biological activity by enriching the sequence with arginine and lysine residues, LPF-35 exhibits improved interactions with negatively charged membrane components and extracellular matrix glycosaminoglycans. In vivo studies demonstrated pronounced analgesic activity, evidenced by significant reduction in nociceptive responses in classic rodent pain models (formalin-induced paw flinch, tail-flick). Its analgesic effects were comparable to that of morphine, but notably, LPF-35 did not induce tolerance or dependence, suggesting a non-opioid mechanism of action—possibly through local anti-inflammatory effects and modulation of nociceptive signaling at nerve endings and ion channels.

Separate experiments, as reported in the patent by Varadhachary et al. [165], used a murine model of chronic skin wounds to assess the topical application of LPF-35 (likely as a gel or cream) at 0.02 mL per wound over 11 days. This treatment significantly accelerated wound healing compared to controls, with improved epithelialization, a higher incidence of complete wound closure, enhanced neovascularization, and a marked reduction in inflammatory cell infiltration. Notably, LPF-35 can be formulated in topical preparations such as polymeric gels. For example, in patent US7420033B2 [165], LPF-35 was incorporated into a vinyl polymer-based hydrogel, enabling stable peptide integration and controlled release without the loss of biological activity.

Although analgesic and regenerative effects of LPF-35 were demonstrated in separate animal models, their combined application in clinical settings, particularly for topical wound treatment, appears to be highly promising. Thus, LPF-35 emerges as a strong candidate for incorporation into next-generation bioactive dressings, aiming to accelerate wound repair while reducing local pain without the need for opioids or NSAIDs. Such an approach could be especially beneficial in the management of chronic wounds, diabetic ulcers, surgical wounds, and burns.

Among endogenous compounds, casomorphins—opioid peptides derived from casein (a milk protein)—are of particular interest, as they have been shown to both modulate pain signaling [166,167] and support wound healing [168]. In this context, Konop et al. [169] developed a keratin-based dressing containing casomorphin (Figure 3), which was applied to full-thickness surgical wounds in mice with iatrogenically induced diabetes. Accelerated healing was observed, including faster re-epithelialization and increased macrophage infiltration at the wound site. Interestingly, keratin fibers from the dressing were incorporated in the regenerating tissue during the healing process.

Importantly, one of the best-known opioid-based peptides with wound-healing activities is dalargin, an enkephalin analog. Several studies have demonstrated its ability to stimulate fibroblast proliferation, enhance capillary formation, promote the development and maturation of granulation tissue and scars, accelerate epithelialization of the wound site, and significantly reduce healing time in rat skin wounds [170,171,172,173]. Although dalargin acts as a μ-opioid receptor agonist, its analgesic effects occur only when administered centrally (e.g., intrathecally or intracerebroventricularly) [174,175]. Peripheral or systemic administration does not produce significant analgesia due to rapid enzymatic degradation and poor BBB penetration. Consequently, dalargin’s antinociceptive activity is limited to central routes of administration, unlike classic opioids such as morphine. As this review focuses on compounds with peripheral analgesic activity, dalargin will not be discussed further.

A structurally and mechanistically distinct compound is the erythropoietin (EPO)-derived peptide ARA 290, an 11-amino acid polypeptide (pEEELERALNSS) [176]. While native EPO exerts both hematopoietic and tissue-protective effects via erythropoietin receptor (EPOR) homodimer activation [177,178], its clinical use beyond anemia is limited by adverse effects related to increased red blood cell production [179,180,181]. In contrast, ARA 290 effectively counteracts capsaicin-induced mechanical hypersensitivity after subcutaneous administration [182] and supports tissue protection and regeneration following injury [183]. For example, ARA 290 significantly alleviated mechanical and cold allodynia in the spared nerve injury (SNI) model in both rats and mice and facilitated regeneration of small autonomic nerve fibers within sympathetic ganglia in diabetic neuropathy [183,184,185,186]. Also, Bohr et al. [187] demonstrated that intravenous administration of ARA 290 beneficially affected microvascular perfusion in a mice model of burn wounds caused by a 10 s application of 90 °C water to the dorsum. Importantly, this was induced via up-regulation of antioxidant response. Likewise, Mashreghi and colleagues [188] showed that this peptide improved the impaired healing process in an STZ-induced mouse model of diabetic foot ulcer (DFU) by decreasing the inflammatory response and then enhancing antioxidant activity.

Given its multifaceted tissue-protective activities, ARA 290 represents a promising candidate for advanced wound care strategies, with future studies warranted to explore localized delivery systems that maximize its therapeutic potential while minimizing systemic exposure.

Delta sleep-inducing peptide (DSIP), composed of the amino acid sequence WAGGDASGG, has also demonstrated notable analgesic and tissue-regenerative effects, though its primary role was promotion of sleep [189]. First isolated in 1977 from rabbit cerebral venous blood, following low-frequency (hypnogenic) electric stimulation to the intralaminar thalamic nuclei [190]. Notably, in 1987, in a study by Yehuda and Carasso [191], DSIP was administered to rats intraperitoneally (i.p.) at doses of 0.1 mg/kg and 1.0 mg/kg. The study demonstrated elevated pain thresholds following DSIP administration in the hot plate test, particularly during the dark phase, and the effect was not blocked by naloxone, indicating a non-opioid analgesic mechanism. However, contradictory results were published by Nakamura et al. [192], who demonstrated DSIP central-induced analgesia blocked by naloxone. As for its efficacy in regenerative medicine, the peptide was suggested to exert wound-healing activities. In this aspect, preliminary steps toward its application in wound dressings have been undertaken, as DSIP was successfully incorporated in two macroporous polymeric matrices: one based on a copolymer of dimethylaminoethyl methacrylate and methylenebisacrylamide (Co-DMAEMA-MBAA), and the other on a copolymer of acrylic acid and methylenebisacrylamide (Co-AA-MBAA) [193].

Neurotensin (NT; pELYENKPRRPYIL; Table 1) is a natural peptide that has been extensively investigated over the years, particularly for its broad therapeutic potential. It has demonstrated strong analgesic activities in various animal models of pain, including those simulating inflammatory and neuropathic conditions [194,195,196]. Moreover, topical application of NT, particularly when delivered via NT-loaded collagen-based dressings, significantly improved wound healing in both control and diabetic C57BL/6 mice. Notably, NT-collagen dressings were more effective in accelerating wound closure in diabetic mice, with observable benefits as early as day 3 post-injury. Those dressings reduced early inflammatory cell infiltration and inhibited extracellular matrix (ECM) degradation mediated by MMP-9. Furthermore, NT-enhanced collagen promoted fibroblast accumulation in granulation tissue and stimulated collagen production and deposition at the wound site, ultimately leading to a more organized and mature collagen matrix [197]. Therefore, owing to its combined analgesic and pro-healing activities, neurotensin holds a strong potential for incorporation into topical peptide-based therapies targeting chronic wounds.

Another peptide worth noting is BPC-157 (GEPPPGKPADDAGLV). This 15-amino acid compound, applied topically as a hydrogel to alkali-burned rat skin, was found to significantly—but dose-dependently—accelerated the wound healing process. Groups of animals treated with BPC-157 showed better granulation tissue formation, re-epithelialization, and remodeling of the dermis as compared to that of the control group, especially on the 18th day after injury [198]. Unfortunately, the results regarding its analgesic effect are inconclusive. For example, BPC-157 did not demonstrate an analgesic effect in an animal model of acute pain with an inflammatory component (the so-called incisional model of pain) [199,200]. On the other hand, clinical data suggest a high analgesic efficacy after intra-articular administration: as many as 87.5% of patients with knee pain experienced improvement following BPC-157 treatment, both as monotherapy and in combination with thymosin beta 4 (TB4) [201,202].

Differences in BPC-157 efficacy between the in vivo animal studies and clinical outcomes in humans are likely to arise from the diversity of pain models used, which do not always fully reflect the complexity and pathomechanisms of human pain. Furthermore, different pharmacokinetic and pharmacodynamic activities of this peptide across species, as well as the method of administration (topical vs. intra-articular), could affect its analgesic effectiveness. Therefore, the animal study results cannot always be directly extrapolated to humans, emphasizing the necessity of further clinical research.

Modern self-assembling formulations have also been developed through conjugation of NSAIDs, such as naproxen, with short-chain peptides (e.g., FFKK). In a study by McCloskey et al. [203], hydrogels were created using the tetrapeptide FFKK and three NSAIDs—ibuprofen, indomethacin, and naproxen—resulting in NSAID-FFKK hybrids (IbuFFKK, IndFFKK, and NpxFFKK) with both anti-inflammatory and antimicrobial activities. It was also shown that the Npx-FFKK conjugate exhibited a greater selectivity for COX-2 and was effective against drug-resistant bacteria responsible for severe hospital-acquired infections.

A natural compound with analgesic and neuroprotective properties (though primarily in the central nervous system) is HCIQ2c1, a peptide isolated from the sea anemone *Heteractis magnifica*, classified as a Kunitz-type serine protease inhibitor [204]. In vitro tests have demonstrated that HCIQ2c1 binds to the open state of the TRPA1 channel, stabilizing it in this form and preventing its transition to a hyperactive closed state [204]. Further experiments showed that intramuscular injection of HCIQ2c1 in mice significantly reduced pain and inflammation induced by allyl isothiocyanate and capsaicin through modulation of TRPA1 and also exerted anti-inflammatory effects by inhibiting intracellular Ca^2+^ release, reactive oxygen species production, pro-inflammatory cytokines, and enzymes involved in arachidonic acid metabolism [204,205].

Crotalphin is a short-chain peptide (sequence: pEFSPENCKGESQPC), an analogue of a natural nociceptive peptide isolated from the venom of the rattlesnake *Crotalus durissus terrificus*. Studies by Konno et al. [206] demonstrated that crotalphin administered orally, intravenously, or via intraplantar injection in rats produced a long-lasting (5-day) antinociceptive effect by activating peripheral KOR opioid receptors in models of mechanical hyperalgesia induced by prostaglandin E2 and carrageenan. A similar study was performed by Gutierrez et al. [207], who demonstrated the peptide to induce a potent and long-lasting opioid antinociceptive effect in neuropathic pain induced in rats by chronic constriction of the sciatic nerve, as it was blocked by naloxone, an opioid receptor antagonist. Importantly, a prolonged crotalphin treatment did not lead to analgesic tolerance [208]. Subsequent research revealed multiple mechanisms behind its antinociceptive effect, including activation of nitric oxide (NO)-dependent cGMP pathways [209], CB2 cannabinoid receptor activation [210], and TRPA1 channel desensitization [211]. However, a recent in vitro study showed that crotalphin might reduce cell viability in primary neuronal cultures [210], which could restrict its clinical applicability.

A relatively recent scientific report highlighted the analgesic potential of melittin (sequence: GIGAVLKVLTTGLPALISWIKRKRQQ), the principal component of bee venom. Subcutaneous administration of melittin at the Zusanli acupoint (ST36) in animals previously treated with paclitaxel, a drug known to induce peripheral neuropathy manifested as cold and mechanical hypersensitivity, significantly reduced both types of hypersensitivity [212]. The analgesic mechanism likely involves endogenous noradrenergic pathways A similar study on rats treated with oxaliplatin also demonstrated that melittin injection at ST36 alleviated mechanical and thermal allodynia. This effect was blocked by intrathecal administration of α1- and α2-adrenergic receptor antagonists, indicating involvement of spinal adrenergic receptors in melittin’s action [213].

Notably, melittin has also been recognized for its antimicrobial activities. Aburayan et al. [214] developed a fibrous material based on polyvinylpyrrolidone (PVP) incorporating melittin fibers. In vitro release tests revealed a complete release of melittin from the PVP matrix within 120 min. Further studies determined the minimum inhibitory concentrations (MICs) for both antibiotic-sensitive and -resistant strains of *S. aureus*, *A. baumannii*, *E. coli*, and *C. albicans*, along with the safe non-cytotoxic melittin concentration. A study by Lima et al. [215] evaluated the antimicrobial and anti-inflammatory efficacy of a melittin-containing ointment applied to non-surgical skin wounds infected with MRSA (*methicillin-resistant Staphylococcus aureus*) in mice. Results showed that melittin’s antimicrobial activity was comparable to that of vancomycin, and the ointment reduced levels of pro-inflammatory cytokines: TNF-α, IL-1β, and IL-6. Another research group developed melittin–diclofenac nanocomplexes (MEL-DCL) for wound treatment, which promoted epithelial regeneration, keratinization, epidermal proliferation, and granulation tissue formation more effectively than either component alone or untreated controls. Treated wounds also showed reduced pro-inflammatory cytokines and increased expression of collagen I alpha 1 (Col1A1), collagen IV alpha 1 (Col4A1) mRNA, and hydroxyproline content. These conjugates also demonstrated antioxidant properties [216]. Similar wound-healing nanocomplexes were developed by combining melittin with copper ions [217] and gabapentin [218].

BN-9 (sequence: YdAGFQPQRF-NH_2_) is a chimeric peptide created by fusing the N-terminal amino acids of biphalin (YdAGFFGAdY) with the C-terminal amino acids of neuropeptide FF (NPFF, FLFQPQRF-NH_2_). It acts as a bifunctional agonist for opioid receptors (MOR, DOR, KOR) and NPFF receptors (NPFF1 and NPFF2). In a formalin test on Kunming mice, Li et al. [219] demonstrated that supraspinal (intracerebroventricular or intrathecal) administration of BN-9 produced stronger antinociceptive effects than morphine. The tail-flick test also showed that BN-9 had a longer-lasting analgesic effect than did morphine. Furthermore, repeated administration of BN-9 retained its efficacy for 8 days without tolerance development, likely owing to simultaneous activation of opioid and NPFF receptors. Interestingly, co-administration of BN-9 with an NPFF receptor antagonist (RF9) led to the development of analgesic tolerance, suggesting that NPFF receptor activation prevented tolerance.

MP-13 (YPFPPVNFKLLSH) is a novel chimeric peptide consisting of morphiceptin (YPFP-NH_2_) and pepcan-9 (PVNFKLLSH). It acts as a bifunctional agonist of opioid and cannabinoid receptors. In a study by Mei et al. [220], MP-13 demonstrated the strongest supraspinal analgesic effect in the mouse tail-flick test following intracerebroventricular injection, outperforming both its precursors and other chimeric analogs. The analgesic mechanism involves simultaneous activation of the MOR, DOR, and CB1 receptors. Importantly, MP-13 did not induce psychological dependence (confirmed by conditioned place preference tests) nor affect gastrointestinal motility—an advantage over classic opioid agonists. During the 7-day treatment schedule, MP-13 maintained stable antinociception without tolerance development, as evidenced by the unchanged microglial cell count in the periaqueductal gray, indicating that chronic exposure did not trigger neuroinflammatory adaptation—a process in which microglia become activated, release pro-inflammatory cytokines, and contribute to opioid tolerance. Opioid-induced microglial activation has been shown to oppose opioid analgesia and enhance tolerance, whereas inhibition or absence of this activation attenuates tolerance development [221,222]. Additionally, MP-13 produced a strong dose-dependent analgesic effect in mouse models of neuropathic, inflammatory, and visceral pains [220].

VF-13, a chimeric peptide combining the pharmacophores of the endogenous cannabinoid peptide VD-hemopressin (α) and neuropeptide FF [223], was pharmacologically evaluated for analgesic activity. Experiments showed that VF-13 activated CB1, NPFF1, and NPFF2 receptors, as evidenced by ERK1/2 phosphorylation, inhibition of forskolin-induced cAMP accumulation, and neurite outgrowth in relevant cell lines (CHO, HEK-293, and Neuro 2A). In an in vivo assay, intracerebroventricular and subcutaneous administration of VF-13 produced dose-dependent antinociception in mouse models of acute and inflammatory pain, primarily via TRPV1 and CB1 pathways. Notably, VF-13 did not induce tolerance over a 6-day treatment period and caused minimum cannabinoid-related side effects such as motor impairment, sedation or gastrointestinal dysfunction [223].

Unfortunately, none of those studies on chimeric peptides assessed their analgesic activity after topical administration.

**Table 1:** Examples of peptides with documented or expected dual antinociceptive and wound-healing activities (amino acids represented by lowercase letters refer to D-configuration, whereas those represented by uppercase letters correspond to L-configuration; p is for pyro).

Peptide	Peptide Sequence	Mechanism of Antinociceptive Activity	Animal Models to Study Antinociceptive Activities	Proregenerative Activity	Experimental Wound Model
Biphalin	YaGF-NH-NH-FGaY	μ and δ opioid receptor agonist	Cancer pain; Acute and inflammatory pain; Neuropathic pain; Visceral pain [157,224,225]	Enhanced healing rate, increased infiltration of macrophages and lymphocytes compared to that of control wounds	Streptozotocin (STZ)-induced diabetic wound [160,161,163]
DALDA	YrFK	peripherally acting μ-opioid receptor (MOR) agonist	Burn injury-induced chronic pain [164]	Not tested	Burn wound [164]
LPF-35	unknown	Unknown, probable opioid receptor-related mechanism	Inflammatory pain models (Formalin paw flinch model; Phenylquinone writhing model; Acetic acid writhing model) [165]	Not tested	Topical/incisional wound [165]
AWL 3106	YaFGYPSaFFGLM	μ and δ opioid receptor agonist/NK-1 (neurokinin 1 receptor agonist)	Acute pain model [226]	Acceleration of the inflammatory phase and promotion of the wound closure	STZ-induced diabetic wound [163]
ARA 290	pEEELERALNSS	Activation of IRR and inhibition of TRPV1 channel	Neuropathic pain models [183,185,227]	Enhancement of wound healing via anti-inflammation, angiogenesis, and collagen deposition; prevention of microvascular thrombosis, thus prevention of the conversion of partial- to full-thickness burn injuries	STZ-induced diabetic wound and burn wound model [187,188]
Neurotensin (NT)	pELYENKPRRPYIL	Agonism at NTS1 and NTS2 receptors	Neuropathic pain models, inflammatory pain models, acute pain model [228,229]	Suppression of inflammatory status, thus promotion of healing	STZ-induced diabetic wound [197]
Melittin	GIGAVLKVLTTGLPALISWIKRKRQQ	activation of spinal α1- and α2-adrenergic receptors	paclitaxel- and oxaliplatin-induced neuropathic pain [212,213]	Antimicrobial activity (in monotherapy), stimulation of epithelial regeneration, keratinization, epidermal proliferation and granulation tissue formation, reduction in proinflammatory cytokine levels, increase in collagen synthesis (in complexes with diclofenac)	Acute wound [216]

It should be emphasized that some of the pain-relieving peptides discussed here (i.e., biphalin, DALDA, and AWL 3106) belong to the opioid family. While this contributes to their strong effectiveness, it also raises concerns regarding the potential for tolerance development and addiction. Hence, there is growing support for the use of compounds that target only peripheral opioid receptors, avoiding the central nervous system, which helps to reduce the risk of common opioid-related side effects.

In this regard, research suggests that prolonged activation of opioid receptors in peripheral tissues (especially in inflammatory conditions) does not necessarily cause tolerance to the analgesic effects [230,231,232]. Although some reports indicate that systemic morphine administration might gradually lose its peripheral anti-inflammatory efficacy [233], a majority of evidence indicates that locally applied opioid agonists can remain safe and effective even during extended treatment. It is believed that this lack of tolerance can be due to the limited systemic absorption and low levels of active metabolites with local delivery. Numerous studies involving patients with chronic wounds, burns, or orthopedic surgeries have confirmed effective localized pain relief accompanied by reduced systemic side effects [234,235].

#### 5.1.2. Peptides with Documented Regenerative Effects—Candidates for Local Analgesia

As mentioned above, none of the actual scientific studies dealing with therapeutic activities of dressings with incorporated analgesic substances directly assess analgesic effects in wound models. This is exemplified by the study of Xiao et al. [236], who analyzed the QHREDGS peptide. In an in vivo model, treatment with a hydrogel containing QHREDGS accelerated wound healing primarily by enhancing re-epithelialization and granulation tissue formation, resulting in a faster wound closure as compared to that of control groups treated with peptide-free gel or standard dressings. Notably, although an increased number of blood vessels was found within the wound, angiogenesis did not significantly change in terms of vessel density or size, suggesting that the beneficial effect of QHREDGS was mainly due to keratinocyte stimulation rather than direct vascular effects. Importantly, pain-related outcomes were not evaluated in this study.

Earlier, in 2010, a 16-amino acid peptide RADA16-I (RADARADARADARADA), capable of spontaneous self-assembly into nanofibers, was reported. In studies on Sprague–Dawley rats with kidney injury, topical application of 2% RADA16-I significantly reduced blood loss [237]. Similarly to QHREDGS, RADA16-I has not yet been evaluated in classical pain models, leaving its potential analgesic activity unconfirmed. However, its ability to modulate inflammation and accelerate tissue regeneration suggests that it could be a promising candidate for future studies aimed at combining regenerative and analgesic therapies.

Other peptidic structures also show promise as pro-regenerative agents. For instance, the h9E peptide, designed by Sun and Huang [238], combines two motifs derived from natural proteins: a spider silk protein motif and a calcium-binding domain from human muscle protein. Applied to a rat tail injury (5 mm incision), h9E exhibited a dose-dependent hemostatic effect with the strongest response at 5% concentration. Its analgesic potential, however, has not yet been assessed.

Promising wound healing stimulation was also reported by a complex of a tripeptide glycyl-L-histidyl-L-lysine (GHK), isolated from human plasma, and copper ions (GHK-Cu). Applied in liposomal form to burn wounds in mice, this complex promoted angiogenesis and shortened healing time. In vitro studies showed that GHK-Cu liposomes enhanced proliferation of human umbilical vein endothelial cells (HUVEC) by increasing the expression of vascular endothelial growth factor (VEGF) and fibroblast growth factor-2 (FGF-2). Notably, analgesic effects have also been found in the mouse hot plate tests, where GHK reduced paw-licking reflexes, indicating pain-relieving activity at very low doses (0.5 μg/kg) [239], though detailed mechanistic descriptions still remain scarce.

Thymosin beta 4 (Tβ4) and tylotoin also exemplify peptides requiring further investigation for analgesic activities, as data regarding pain modulation are currently lacking. Tβ4, a natural 43-amino acid water-soluble peptide present in all tissues except red blood cells, has shown in vivo efficacy in accelerating wound healing. Application as a hydrogel [202] or chitosan-collagen dressing [240] sped wound closure, increased collagen deposition, and enhanced angiogenesis. Intraperitoneal Tβ4 administration following lipopolysaccharide (LPS) injection reduced pro-inflammatory cytokines in mouse plasma [241]. However, clinical trials with Tβ4 gels at various concentrations (0.01, 0.02, 0.03, and 0.1%) in patients with pressure ulcers, venous leg ulcers, and epidermolysis bullosa showed no statistically significant difference in healing as compared to that of placebo, although some trends towards faster healing were noted at intermediate doses during early weeks [242,243].

Tylotoin is a 12-amino-acid peptide with the sequence KCVRQNNKRVCK, derived from the skin of the salamander *Tylototriton verrucosus* [244]. It has been found to stimulate wound healing through proliferation of keratinocytes, vascular endothelial cells, and fibroblasts, with an effect comparable to that of epidermal growth factor. This results in accelerated re-epithelialization and granulation tissue formation at the wound site [244]. Unfortunately, its susceptibility to enzymatic degradation significantly limits its medical applicability. To address this, a research team led by Wang et al. [245] developed nanoparticles based on chitosan and a copolymer of lactic and glycolic acids, enabling sustained release of tylotoin over a period of 14 days. These nanoparticles were applied topically to wounds in mice. The combination of tylotoin and chitosan not only enhanced wound healing but also provided antibacterial and hemostatic activities, attributed to chitosan. Furthermore, it was shown that wounds treated with tylotoin-loaded nanoparticles only once in every two weeks exhibited superior scarless healing as compared to wounds treated with daily tylotoin application.

Another naturally derived peptide is OA-GL12, a short 12-amino-acid peptide (sequence: GLLSGINAEWPC) isolated from the skin of the frog *Odorrana andersonii*. In an in vivo study on full-thickness wounds in mice, it demonstrated wound-healing activity by inducing secretion of TNF and TGF-β1 and by scavenging free radicals [246].

A wound dressing was also developed using a hyaluronic acid-based hydrogel containing peptides Pep4 (KRCCPDTCGIKCL) and Pep4M (KRMMPDTMGIKML) [247]. Pep4 is a selected fragment of SLPI (Secretory Leucocyte Protease Inhibitor), whereas Pep4M is a modified form in which the highly reactive cysteine residues were replaced with less reactive methionine residues. Both peptides show moderate elastase inhibitory activity. Under physiological conditions, elastase plays an important role in wound healing by degrading damaged tissue and enabling full regeneration, but its excess can disrupt the balance between tissue degradation and remodeling [248,249,250]. It has been shown that hydrogel dressings containing low concentrations of Pep4 and Pep4M can support wound healing and tissue regeneration. An interesting finding was an increased elastase-inhibitory activity of Pep4 in the presence of reactive oxygen species, which could be particularly useful in inflammatory conditions associated with elevated oxidative stress [247].

β-neoendorphin (β-NEP) is an endogenous opioid peptide (sequence: YGGFLRKYP) discovered in the 1980s. It is formed through enzymatic cleavage of prodynorphin. Studies in rats have shown that the highest concentrations of β-NEP were found in the median eminence of the brain [251]. One [252] of the few studies on β-NEP, conducted in vitro on human HaCaT keratinocytes, reported that β-NEP induced migration of keratinocytes toward the wound site (a scratch wound was created on the monolayer with a pipette tip). This migration was found to be triggered by activation of the MAPK/Erk1/2 signaling pathway. In the same study, a similar experiment was conducted using mouse embryonic fibroblasts, and cell migration toward the wound was also reported. Interestingly, fibroblast migration under the influence of β-neoendorphin was not inhibited even in the presence of mitomycin C, a popular proliferation blocker [252].

Importantly, the lack of direct analgesic assessments highlighted in this section reflects a broader research paradigm in which regenerative efficacy is prioritized, while pain-related endpoints are rarely incorporated into study designs. This imbalance stems from a methodological focus on structural and histological outcomes rather than from evidence arguing against pain relevance.

At the biological level, however, tissue repair and pain regulation are closely interconnected. Processes such as modulation of inflammatory signaling, control of oxidative stress, and restoration of tissue integrity inherently influence the local nociceptive environment, even when analgesia is not an explicit therapeutic goal. Consequently, the current body of evidence should not be interpreted as negative with respect to analgesic potential, but rather as incomplete. The regenerative effects documented across studies provide a strong rationale for expanding future experimental frameworks to include systematic evaluation of pain-related outcomes, enabling the development of therapeutic strategies that integrate tissue regeneration with functional pain modulation.

### 5.2. Application and Effectiveness of Peptide Structures in the Treatment of Wound Pain—Clinical Trials Outcomes

Despite the demonstrated analgesic activity of selected peptides in animal models, no reliable clinical data are currently available regarding the use of short-chain peptides for treatment of wound-related pain in human patients. Previous experimental studies were primarily focused on the management of neuropathic or cancer-related pain, where analgesic peptides were administered via injection (e.g., intrathecal or intracerebroventricular), or on the stimulation of wound healing processes, where the peptides facilitated tissue regeneration mechanisms.

At the same time, analgesia-focused studies were largely concentrated on central mechanisms of action, with opioid receptors serving as the principal pharmacological targets. However, discoveries from the 1990s shed new light on the possibility of targeting peripheral opioid receptors. It was then demonstrated that the receptors were also expressed at peripheral terminals of sensory nerve fibers in the skin. Although opioids show rather poor peripheral effects under normal physiological conditions, their activity becomes evident shortly after induction of inflammation [253,254].

The success of topically applied morphine in clinical settings [128,129] suggests that direct application of opioid-like analgesic peptides onto wounds can produce local anesthesia. Preclinical studies in animals have shown that opioid peptides do not induce central side effects [255,256,257], positioning them as a potentially effective and safe alternative to conventional analgesics—many of which (e.g., diclofenac) impair wound healing or have a limited systemic penetration but still carry a risk of central nervous system (CNS) side effects (as is the case with regular opioids).

Furthermore, active research is ongoing into the development of analgesic peptides acting through non-opioid mechanisms. Promising pharmacological targets include NK-1, TRPV1, NPFF1, and CB1 receptors [258,259]. An intriguing strategy involves the combined application of two peptides with distinct roles played in the wound healing process.

Given the complementary mechanisms of activity of analgesic and pro-regenerative peptides, it is reasonable to hypothesize a potential synergistic effect. The co-administration of peptides with analgesic and regenerative properties can enhance both the anti-nociceptive and anti-inflammatory outcomes. However, to confirm this hypothesis, extensive in vivo studies in animal models and clinical trials involving patients with chronic, painful wounds are required. Additionally, there is a pressing need to develop reliable and reproducible methods for evaluating analgesic efficacy in in vivo wound models. Since this study focuses on wound-associated pain, a biochemical approach, such as quantifying levels of pain mediators (e.g., substance P) in body fluids at defined time points, appears to be the most desirable method. Nevertheless, alternative or complementary testing methods are still missing, restricting the interpretability and reproducibility of the outcomes.

## 6. Limitations and Challenges Related to the Use of Peptides Incorporated in Wound Dressings

Despite their promising therapeutic potential, peptide-based wound dressings face a number of scientific, technical, regulatory, and clinical limitations that hinder their widespread adoption in clinical practice. These challenges affect both the design and performance of peptide-integrated systems, as well as their scalability and long-term safety in wound management.

One of the primary challenges in incorporating peptides into wound dressings is their inherent chemical and enzymatic instability. Peptides are susceptible to degradation by proteases occurring in the wound microenvironment, especially in chronic and infected wounds where protease activity is significantly elevated [260]. This leads to reduced therapeutic efficacy and a short duration of action unless stabilization strategies have been employed.

To overcome this, various techniques such as peptide cyclization, amino acid substitution with D-isomers, PEGylation, and encapsulation in carriers (e.g., nanoparticles, hydrogels) are being explored, though these approaches can increase complexity and production costs. Indeed, since proteolytic enzymes prefer the natural L-configuration rather than the D-one, it is well-documented that replacing L-amino acids with their D-enantiomers, incorporating methylated amino acids, or introducing β-amino acids and peptoid residues are among the usual approaches to prolonging the plasma half-life. An illustrative example is selepressin, a synthetic analog of vasopressin, which preserves receptor specificity while exhibiting a substantially longer half-life in comparison to that of the native peptide [261,262]. Also, the opioid peptide metkephamid, an analog of the methionyl-enkephalin, owing to the incorporation of D-alanine, had a half-life of approx. 60 min vs. seconds in the case of its mature compound [263]. Similar findings are for PEGylation, where polyethylene glycol chains are covalently attached to the peptide backbone. This modification increases molecular size, reduces renal clearance, and shields the peptide from enzymatic degradation [264]. A prominent example is hematide, a dimeric peptide, known also as a PEGylated peptide-based erythropoiesis stimulating agent [265]. It has half-lives substantially longer than those published for the recombinant human erythropoietin rHuEPO (2.5 h) [266].

In addition to the just-mentioned facts, achieving controlled and sustained release of active peptides at the wound site remains a significant technological challenge, particularly in maintaining consistent therapeutic concentrations over time. Peptides tend to diffuse rapidly from hydrophilic wound dressings, leading to a burst release profile and suboptimum exposure [267]. Advanced biomaterials and smart delivery systems (e.g., pH-sensitive matrices or stimuli-responsive hydrogels) are under investigation to address this, but their translation to clinically approved products remains still restricted.

Although peptides are generally considered to be less immunogenic than proteins, the repeated or prolonged exposure of damaged tissue to synthetic or modified peptides can still pose a risk of local irritation, allergic response, or immune activation [268,269,270]. This is particularly relevant in patients with compromised immune function or chronic inflammation, where even low-level immunogenicity can lead to anti-drug antibody (ADA) formation and decreased therapeutic efficacy [271]. One of the key contributors to the immunogenic potential of therapeutic peptides lies in the impurities introduced during chemical synthesis and manufacturing. In solid-phase peptide synthesis (SPPS), even minor contaminants present in the starting amino acids, such as dipeptides, D-isomers, β-alanine, or structurally altered residues, can become unintentionally integrated into the final peptide product. Moreover, limitations inherent to the SPPS method can generate aberrant sequences, including incomplete chains, internal deletions, duplicated or misincorporated amino acids, and racemized residues. Post-synthetic modifications, including chemical alterations of terminal groups or side chains due to insufficient deprotection, contribute to heterogeneity as well [270,272].

In addition, as just mentioned, peptides are prone to chemical degradation over time, especially under suboptimum formulation conditions such as inappropriate pH, elevated storage temperatures, or prolonged storage. The degradation can lead to oxidative or reductive changes in amino acid side chains or interactions with formulation excipients, potentially generating immunogenic breakdown products. These alterations not only compromise therapeutic efficacy and stability but can also introduce non-native structures that elicit immune responses [270,273]. For this reason, rigorous control of synthesis quality and stability profiling is critical to minimizing adverse immunological outcomes.

An important consideration regarding peptide-functionalized wound dressings is their frequent classification as combination products, comprising both a medical device and a biologically active compound. This consequently subjects them to rigorous and often lengthy regulatory processes. Regulatory agencies such as the Food and Drug Administration (FDA) and European Medicines Agency (EMA) demand extensive documentation on pharmacodynamics, pharmacokinetics, stability, biocompatibility, and potential immunogenicity [274,275,276]. These requirements can delay market entry and discourage commercial investment.

In summary, while peptide-based wound dressings offer significant promise owing to their targeted biological activity and potential to enhance healing, numerous scientific, technical, and regulatory challenges still remain to be addressed before their full clinical potential can be realized. Advances in peptide stabilization, delivery technologies, and manufacturing processes, combined with streamlined regulatory pathways and thorough immunogenicity assessments, will be critical to overcoming current limitations. Continued interdisciplinary research and collaboration between academia, industry, and regulatory bodies are essential to translate these innovative materials from bench to bedside, ultimately improving outcomes for patients with acute and chronic wounds.

## 7. Conclusions

Wound-related pain remains a major clinical challenge, as it not only affects patients’ quality of life but also interferes with the healing process. Conventional analgesic strategies, though effective, are often associated with systemic side effects, impaired tissue repair, or limited efficacy in chronic wounds. In this context, peptide-based therapeutics emerge as a promising alternative, offering the unique potential to combine localized analgesic activity with regenerative and anti-inflammatory characteristics. Preclinical evidence demonstrates that several natural and synthetic peptides—such as biphalin, LPF-35, neurotensin, or ARA 290, can simultaneously modulate nociceptive pathways and accelerate tissue repair.

Incorporation of those peptides into advanced wound dressings represents a particularly attractive strategy, enabling sustained release at the site of injury, reducing systemic exposure, and enhancing patient comfort. However, challenges including peptide instability, proteolytic degradation, potential immunogenicity, and regulatory complexity still limit their widespread clinical application. Future research should therefore focus on the development of stable formulations, reliable preclinical models that integrate both analgesic and regenerative endpoints, and well-designed clinical trials to validate efficacy and safety in human patients. Importantly, new experimental approaches are also needed to directly evaluate the analgesic activity of peptides in wound models, as current methods rarely address pain originating specifically from damaged skin.

Taken together, dual-action peptide-based therapies hold the potential to transform wound management by bridging the gap between effective pain relief and accelerated tissue regeneration, ultimately improving therapeutic outcomes in both acute and chronic wounds.

## Data Availability

No new data were created or analyzed in this study. Data sharing is not applicable to this article.
